# Asthma Therapies on Pulmonary Tuberculosis Pneumonia in Predominant Bronchiectasis–Asthma Combination

**DOI:** 10.3389/fphar.2022.790031

**Published:** 2022-03-30

**Authors:** Jun-Jun Yeh, Hui-Chuan Lin, Yu-Cih Yang, Chung-Y. Hsu, Chia-Hung Kao

**Affiliations:** ^1^ Department of Family Medicine, Geriatric Medicine, Chest Medicine and Medical Research, Ditmanson Medical Foundation Chia-Yi Christian Hospital, Chiayi, Taiwan; ^2^ College of Medicine, China Medical University, Taichung, Taiwan; ^3^ Department of Pharmacy, Ditmanson Medical Foundation Chia-Yi Christian Hospital, Chiayi, Taiwan; ^4^ Management Office for Health Data, China Medical University Hospital, Taichung, Taiwan; ^5^ College of Medicine, China Medical University, Taichung, Taiwan; ^6^ Graduate Institute of Biomedical Sciences, College of Medicine, China Medical University, Taichung, Taiwan; ^7^ Department of Nuclear Medicine and PET Center, China Medical University Hospital, Taichung, Taiwan; ^8^ Department of Bioinformatics and Medical Engineering, Asia University, Taichung, Taiwan; ^9^ Center of Augmented Intelligence in Healthcare, China Medical University Hospital, Taichung, Taiwan

**Keywords:** pulmonary tuberculosis, pneumonia, bronchiectasis‐asthma combination, beta2 agonist/muscarinic antagonists, steroids, and benzodiazepines

## Abstract

**Background:** It is sometimes difficult to distinguish between asthma and bronchiectasis as their symptoms overlap, and these two diseases are associated with pulmonary tuberculosis (PTB) or pneumonia.

**Objective:** The purpose of this study is to determine the effects of bronchodilator drugs, steroids, antidepressants drugs, and antianxiety drugs on the risks of PTB or pneumonia in patients with bronchiectasis–asthma combination or bronchiectasis–asthma–chronic obstructive pulmonary disease combination—BCAS cohort.

**Methods:** After propensity score matching, we retrospectively studied patients with BCAS (*N* = 620) and without BCAS (*N* = 2,314) through an analysis. The cumulative incidence of PTB or pneumonia was analyzed through Cox proportional regression. After adjustment for sex, age, comorbidities, and medications [including long-acting beta2 agonist/muscarinic antagonists (LABAs/LAMAs), short-acting beta2 agonist/muscarinic antagonists (SABAs/SAMAs), leukotriene receptor antagonist, montelukast, steroids (inhaled corticosteroids, ICSs; oral steroids, OSs), anti-depressants (fluoxetine), and anti-anxiety drugs (benzodiazepines, BZDs)], we calculated the adjusted hazard ratios (aHR) and their 95% confidence intervals (95% CI) for these risks. Similar to OSs, ICSs are associated with an increased risk of PTB or pneumonia, lumping these two as steroids (ICSs/OSs).

**Results:** For the aHR (95% CI), with non-LABAs/non-OSs as the reference 1, the use of LABAs [0.70 (0.52–0.94)]/OSs [0.35 (0.29–0.44)] was associated with a lower risk of PTB or pneumonia. However, the current use of LABAs [2.39 (1.31–4.34)]/SABAs [1.61 (1.31–1.96)], steroids [ICSs 3.23 (1.96–5.29)]/OSs 1.76 (1.45–2.14)], and BZDs [alprazolam 1.73 (1.08–2.75)/fludiazepam 7.48 (1.93–28.9)] was associated with these risks. The current use of LAMAs [0.52 (0.14–1.84)]/SAMAs [1.45 (0.99–2.11)] was not associated with these risks.

**Conclusion:** The current use of LAMAs/SAMAs is relatively safe with respect to PTB or pneumonia risks, but LABAs/SABAs, steroids, and BZDs could be used after evaluation of the benefit for the BCAS cohort. However, we must take the possible protopathic bias into account.

## Introduction

Asthma is an inflammatory and immune-related disease. Bronchiectasis is a chronic, progressive, infective, immune-related, and inflammatory obstructive airway disease (Martínez-García et al., 2018). Meanwhile, bronchiectasis, in general, results from airway damage from previous infection(s), perhaps mediated by elastase activity. Bronchiectasis complicating asthma is associated with the above-stated asthma phenotypic expression. There also is a more proximal, cylindrical bronchiectasis associated with allergic bronchopulmonary aspergillosis (ABPA) complicating asthma and with an eosinophilic phenotype. Moreover, asthma may be linked with bronchiectasis (BCAS), especially in cases of steroid-resistant asthma (Polverino et al., 2018). Certainly, the treatment of patients with BCAS is related somewhat to the treatment of asthma without bronchiectasis, and the treatment of bronchiectasis without asthma differs considerably.

BCAS is associated with frequent hospitalizations, concomitant gastroesophageal reflux disease, hypersensitivity to non-steroidal anti-inflammatory drugs, and high blood eosinophil counts and represents an additional phenotypic feature of severe eosinophilic asthma (Truong, 2013; Coman et al., 2018). In precision and personalized medicine, BCAS is considered a different entity from asthma (Polverino et al., 2018). One study indicated that the coronavirus predisposed patients to bronchiectasis or the exacerbation of asthma (Zhang et al., 2020). The coronavirus may play a role in the development of BCAS (Zhang et al., 2020). Thus, BCAS is an essential topic in the era of the coronavirus.

One study indicated that previous pneumonia, pulmonary tuberculosis (PTB), and non-tuberculous mycobacterium (NTM) increased the risk of bronchiectasis (Hill et al., 2019). Similar to oral steroid (OS) users, inhaled corticosteroid (ICS) users have a higher risk of PTB or pneumonia in asthma or chronic obstructive pulmonary disease (COPD) (Chang et al., 2016; Lee et al., 2019; Yeh et al., 2020). Meanwhile, PTB or pneumonia may coexist when steroids (ICSs/OSs) are used among BCAS (Feng et al., 2012; Chang et al., 2016; Yeh, 2019; Yeh et al., 2020). Few studies have investigated the relationship between PTB, pneumonia, bronchodilators, steroids, leukotriene receptor antagonists, and montelukast among a BCAS cohort. Moreover, no study focuses on the impact of antidepressants and benzodiazepines (BZDs) for PTB or pneumonia among the BCAS cohort. We wanted to evaluate the effects asthma therapies have on risk of PTB or pneumonia among the BCAS cohort by examining the general population. Meanwhile, protopathic bias occurs when a drug is prescribed for initial signs/symptoms of an outcome not yet diagnosed, reflecting a reversal of cause and effect (Arfè and Corrao, 2016). This type of bias is likely to arise in studies on the associations between drug use and PTB risk (studying outcomes with long latencies). We added this bias into account in this study.

## Materials and Methods

### Data Source

Data for this retrospective, population-based cohort study were obtained from the National Health Insurance Research Database (NHIRD), which was launched in 1995 and covers almost 99% of the 23 million people in Taiwan. We used the Longitudinal Health Insurance Database 2000, which maintains the registration data of everyone who was a beneficiary of the National Health Insurance program during the period of 1996–2000. Data on the medical services provided by programmers are collected by the National Health Insurance Administration and entered into the NHIRD. This longitudinal database contains population-level demographic and administrative information, including sex, birth year, region of residence, dates of admission and discharge, prescription drugs, surgical procedures, and diagnostic codes. The International Classification of Diseases Ninth Revision, Clinical Modification (ICD-9-CM) diagnostic codes are also listed, with a maximum of five codes. In the NHIRD, the ICD-9-CM and procedure coding system are used to define the diagnostic and procedure codes, respectively. In accordance with the Personal Information Protection Act, individual identifiers are encrypted before release for research. The NHIRD has been used for various studies and provides high-quality information on diagnoses, hospitalizations, and prescriptions.

### Ethics Statement

The NHIRD encrypts personal information to protect patients’ privacy. It provides researchers with anonymous identification numbers associated with relevant claims information, including sex, date of birth, medical services received, and prescriptions. Therefore, patient consent is not required to access the NHIRD. The study protocol was approved by the Institutional Review Board of China Medical University (CMUH104-REC2-115-AR4), which also specifically waived the informed consent requirement.

### Study Population

Supplementary Figure S1 shows the process of selecting participants for study cohorts. We identified patients diagnosed with new bronchiectasis (ICD-9-CM code 494) or with new COPD (ICD-9-CM codes 491, 492, and 496) from claims data for 2000 to 2012. Patients aged ≧18 years having new bronchiectasis + new asthma (ICD-9-CM code 493) or new bronchiectasis + new asthma + new COPD were selected for the BCAS cohort. The comparison cohort (non-BCAS cohort) was randomly selected from the rest of the bronchiectasis, COPD, asthma, or patients with immunosuppressants such as steroid use who are without a diagnosis of BCAS.

To evaluate the risk of PTB or pneumonia in patients within the BCAS cohort, we selected a cohort of patients who had two outpatient visits or an inpatient visit for the BCAS cohort and a comparable non-BCAS cohort (Redondo et al., 2016; Vanfleteren, 2016; Hung et al., 2018; Mihălț;an and Constantin, 2019; Veith et al., 2020).

The BCAS cohort includes six bronchiectasis + asthma (pure BCAS) and seven bronchiectasis + asthma + COPD (BCAOS). The non-BCAS cohort (without bronchiectasis–asthma combination or without bronchiectasis–asthma–COPD combination) includes 1 pure bronchiectasis = (1 + 4 + 6 + 7, new bronchiectasis) − 4 BCOS − 6 BCAS − 7 BCAOS; 2 pure COPD = (2 + 4 + 5 + 7, new COPD) − 4 BCOS − 5 ACOS − 7 BCAOS; 3 pure asthma = (3 + 5 + 6 + 7, new asthma) − 5 ACOS − 6 BCAS − 7, BCAOS; 4 bronchiectasis + COPD (BCOS); 5 COPD + asthma (ACOS); and 8 others—such as patients with steroids use. The exclusion criteria include 1) diagnoses of PTB (ICD-9-CM codes 010–012) and pneumonia (codes 480–486) before entry into the study, wash out period 1996–1999; 2) age <18 years; and 3) incomplete demographic information (Supplementary Figure S1) (Shantakumar et al., 2018). We identified patients in the newly diagnosed BCAS cohort (*N* = 664) and the newly diagnosed non-BCAS cohort (*N* = 694,576) between 1 January 2000 and 31 December 2012.

These patients in the BCAS cohort were matched to patients in the non-BCAS cohort by age (5-year range), gender, comorbidities, smoking status, medications, and year of index date according to incidence density sampling. After 1:4 matching, the BCAS cohort (*N* = 620) and the non-BCAS cohort (*N* = 2,314) were found (Supplementary Figure S1) (Redondo et al., 2016; Vanfleteren, 2016; Veith et al., 2020).

The primary study outcome was the occurrence of PTB or pneumonia (outpatient and inpatient settings: PTB: ICD-9-CM codes 010–012; pneumonia: codes 480–486). Both cohorts were observed from the index date to the date of diagnosis of (PTB or pneumonia), withdrawal from the National Health Insurance program, or to the end of 2013, depending on whichever occurred first. Patients were followed up until death or the end of the study period (31 December 2013).

The incidence of PTB or pneumonia was defined as a new diagnosis and at least one visit with inpatient claims data or two visits with outpatient claims data after the index date. The ICD-9 CM, Anatomical Therapeutic Chemical (ATC), and full names list are presented in Supplementary Table S1. This study adjusted for the demographic factors of gender and age, comorbidities, and medications. Those comorbidities were calculated on the basis of the participants’ status before the index date. We defined the index date of the case cohort by the first date of drug prescription after a diagnosis of BCAS, and we restricted the case cohort to patients who used drugs for more than 28 days.

### Statistical Analysis

Propensity score matching was used to balance the groups with respect to the known variables and to increase their comparability. Although some unmeasurable confounders may exist disproportionally between the study groups, propensity score matching can optimally balance the distributions of measured covariates as much as a randomized trial does. We estimated the patients’ propensity scores through a non-parsimonious multivariable logistic regression, with receipt of patients with or without BCAS as the independent variable. We incorporated clinically relevant covariates (comorbidities, medications, etc.) into our analysis—the primary analysis. PTB or pneumonia was the dependent variable (Table 1; Supplementary Figure S2). We used Student’s *t* test and the chi-square test to compare differences in baseline characteristics and comorbidities between the BCAS cohort and non-BCAS cohort. The incidence density rate (per 1,000 person-years) was analyzed to estimate the incidence of PTB or pneumonia among the cohorts stratified by gender, age, comorbidities, and medications. The annual incidence density rate was calculated by dividing the number of new diagnoses of PTB or pneumonia by the number of person-years for patients at risk for PTB or pneumonia for each year between 2000 and 2013. The risks of PTB or pneumonia between these two cohorts were compared through Cox proportional hazard regression models. The analysis was adjusted for gender, age, comorbidities, and medications for the BCAS cohort. We set our significance threshold at *α* = 0.05 for *a priori* hypotheses. Significance in subsequent explanatory analyses was defined as *p* < 0.01. Statistical analysis was performed using SAS (version 9.4 for Windows; SAS Institute, Inc., Cary, NC, United States).

**TABLE 1:** Baseline characteristics of study population.

Variable	Original population				*p*-value^a^	PS-matching population				*p*-value^a^
	BCAS cohort (*n* = 664)		Non-BCAS cohort (*n* = 694,576)			BCAS cohort (*n* = 620)		Non-BCAS cohort (*n* = 2,314)		
	*N*	%	*N*	%		*N*	%	*N*	%	
Gender					0.0003					0.40
Female	372	56.0	340,724	49.1		345	55.6	1,244	53.8	
Male	292	44.0	353,852	50.9		275	44.4	1,070	46.2	
Age at baseline, years					<0.0001					0.36
<35	69	10.4	339,290	48.9		65	10.5	215	9.29	
≥35	595	89.6	355,286	51.1		555	89.5	2099	90.7	
Mean (SD)^b^	59.1 (17.7)		36.8 (18.2)		<0.0001	58.8 (17.7)		60.9 (18.0)		0.001
Comorbidity										
Non-tuberculous mycobacterium	1	0.15	139	0.02	0.01	1	0.16	9	0.39	0.38
Rheumatoid arthritis	24	3.61	7,269	1.05	<0.0001	19	3.06	92	3.98	0.29
Diffuse connective disease and Sjogren’s Syndrome	21	3.16	5,946	0.86	<0.0001	18	2.90	83	3.59	0.40
COPD	364	54.8	26,154	3.77	<0.0001	336	54.1	1,271	54.9	0.74
Diabetes	186	28.0	66,474	9.57	<0.0001	171	27.5	696	30.0	0.22
Aspergillosis	1	0.15	19	0.002	<0.0001	1	0.16	4	0.17	0.95
Candidiasis	0	0	10	0.001	0.92	0	0	0	0	—
Endemic mycoses	0	0	43	0.01	0.83	0	0	0	0	—
Mounier-Kuhn	0	0	37	0.01	0.85	0	0	0	0	—
Cystic fibrosis	0	0	4	0.001	0.95	0	0	0	0	—
Hypertension	328	49.4	95,776	13.8	<0.0001	298	48.0	1,206	52.1	0.07
Hyperlipidemia	163	24.5	61,673	8.88	<0.0001	151	24.3	613	26.4	0.28
Pulmonary embolism	0	0	173	0.02	0.68	0	0	0	0	—
Depression	14	2.11	4,608	0.66	<0.0001	13	2.10	56	2.42	0.63
Stroke	62	9.34	14,928	2.15	<0.0001	54	8.71	245	10.5	0.16
Heart disease	271	40.8	59,964	8.63	<0.0001	247	39.8	1,008	43.5	0.09
Anxiety	219	32.9	70,332	10.1	<0.0001	196	31.6	770	33.2	0.43
Smoking										
Tobacco dependence	3	0.45	747	0.11	0.006	3	0.48	13	0.56	0.81
Tobacco use disorder complicating pregnancy	0	0	0	0	—	0	0	0	0	—
Medication										
LABA	131	19.7	806	0.12	<0.0001	117	18.8	278	12.0	<0.0001
LAMA	38	5.72	595	0.09	<0.0001	32	5.16	81	3.50	0.05
SABA	231	34.8	16,880	2.43	<0.0001	216	34.8	748	32.3	0.23
SAMA	150	22.6	11,876	1.71	<0.0001	140	22.5	510	22.0	0.77
ICSs	196	29.5	1,250	0.18	<0.0001	177	28.5	437	18.8	<0.0001
OSs	608	91.6	510,508	73.5	<0.0001	567	91.4	2,140	92.4	0.39
Leukotriene antagonist	57	8.58	1,426	0.21	<0.0001	52	8.39	100	4.32	<0.0001
Montelukast	55	8.28	1,401	0.20	<0.0001	50	8.06	99	4.28	0.0001
Alprazolam	224	33.7	98,484	14.1	<0.0001	203	32.7	848	36.6	0.07
Fluoxetine	0	0	271	0.04	0.61	0	0	0	0	—
Fludiazepam	112	16.9	39,967	5.75	<0.0001	103	16.6	393	16.9	0.82

a *p*-value using chi-square for the comparisons between with and without BCAS cohort.

BCAS cohort: bronchiectasis–asthma combination cohort; comparison cohort: non-BCAS cohort.

b Average age using Wilcoxon rank-sum test for verification.

COPD, chronic obstructive pulmonary disease; LABAs/LAMAs: long-acting beta2 agonist/muscarinic antagonist; SABAs/SAMAs, short-acting beta2 agonist/muscarinic antagonist; ICSs, inhaled corticosteroids; OSs, oral steroids.

## Results

### Baseline Characteristics

No significant difference in the distribution of age groups was observed between the two cohorts because of the success of the incidence density sampling (Table 1). The mean age (standard deviation) of the participants was 58.8 (±17.7) years for the BCAS cohort and 60.9 (±18.0) years for the non-BCAS cohort (Student’s *t* test: *p* < 0.001). The majority of patients were more than 35 years old. Several significant differences in gender, age, grouped comorbidities, and grouped medications were observed between the BCAS and non-BCAS cohorts; the use of LABAs, ICSs, OSs, leukotriene receptor antagonist, and montelukast was more frequent in the BCAS cohort than in the non-BCAS cohort.

### Comparison of PTB or Pneumonia Risks Among Groups With Non-Comorbidity and Non-Medicine Use as Reference

The incidence density rate of PTB or pneumonia was higher in the BCAS cohort than in the non-BCAS cohort (57.7 and 46.7 per 1,000 person-years, respectively) (Table 2). These results reveal that the risk of developing PTB or pneumonia was 1.54 times higher for the BCAS cohort [95% confidence interval (CI) = 1.31–1.80) than for the non-BCAS cohort. Age of over 35 years, male sex, diabetes, stroke, and heart disease were significantly and positively associated with PTB or pneumonia. Comorbidities of hyperlipidemia and depression and the use of LABAs, OSs, and BZDs were significantly and negatively associated with PTB or pneumonia. The risks of PTB or pneumonia were 1.18-fold and 2.64-fold higher for patients older than 35 years of age and men, respectively, than for patients younger than 35 years of age (95% CI = 1.03–1.36 and 1.90–3.68). The risks of PTB or pneumonia were significantly higher for those with diabetes [adjusted hazard ratio (aHR) = 1.19, 95% CI = 1.03–1.37), stroke (aHR = 1.36, 95% CI = 1.13–1.64), and heart disease (aHR = 1.19, 95% CI = 1.03–1.38). The risks were significantly lower for those with hyperlipidemia (aHR = 0.75, 95% CI = 0.64–0.88); depression (aHR = 0.53, 95% CI = 0.30–0.92); and who use LABAs (aHR = 0.70, 95% CI = 0.52–0.94), OSs (aHR = 0.35, 95% CI = 0.29–0.44), alprazolam (aHR = 0.73, 95% CI = 0.63–0.85), and fludiazepam (aHR = 0.79, 95% CI = 0.66–0.95). However, the effects of other variables (Table 2) were not found to be significant.

**TABLE 2:** Cox model-measured hazard ratios and 95% confidence interval of pulmonary tuberculosis or pneumonia associated with gender, age, comorbidity, and medications after propensity matching of both the BCAS and non-BCAS cohorts.

	Pulmonary tuberculosis or pneumonia			Crude HR (95% CI)	Adjusted HR (95% CI)
	Event	PY	IR		
Bronchiectasis–asthma					
No	743	15,909	46.7	1 (reference)	1 (reference)
Yes	208	3,602	57.7	1.23 (1.05–1.43)**	1.54 (1.31–1.80)***
Gender					
Female	470	10,515	44.7	1 (reference)	1 (reference)
Male	481	8,996	53.5	1.19 (1.05–1.36)**	1.18 (1.03–1.36)*
Age					
<35	41	2,558	16	1 (reference)	1 (reference)
≥35	910	16,953	53.7	3.46 (2.53–4.74)***	2.64 (1.90–3.68)***
Comorbidity					
Non-tuberculous mycobacterium					
No	950	19,451	48.8	1 (reference)	1 (reference)
Yes	1	60	16.7	0.34 (0.04–2.45)	0.63 (0.08–4.51)
Rheumatoid arthritis					
No	915	18,852	48.5	1 (reference)	1 (reference)
Yes	36	659	54.6	1.13 (0.81–1.58)	1.03 (0.73–1.45)
Diffuse connective disease and Sjogren’s syndrome					
No	919	19,003	48.4	1 (references)	1 (reference)
Yes	32	508	63	1.32 (0.93–1.88)	1.18 (0.82–1.70)
COPD					
No	372	9,990	37.2	1 (reference)	1 (reference)
Yes	579	9,521	60.8	1.67 (1.46–1.90)***	1.15 (0.99–1.32)
Diabetes					
No	613	14,439	42.5	1 (reference)	1 (reference)
Yes	338	5,072	66.6	1.59 (1.39–1.82)***	1.19 (1.03–1.37)*
Aspergillosis					
No	950	19,485	48.8	1 (reference)	1 (reference)
Yes	1	26	38.5	0.79 (0.11–5.65)	1.05 (0.14–7.52)
Candidiasis					
No	951	19,511	48.7	1 (reference)	1 (reference)
Yes	0	0	0	—	—
Endemic mycoses					
No	951	19,511	48.7	1 (reference)	1 (reference)
Yes	0	0	0	—	—
Mounier-Kuhn					
No	951	19,511	48.7	1 (reference)	1 (reference)
Yes	0	0	0	—	—
Cystic fibrosis					
No	951	19,511	48.7	1 (reference)	1 (reference)
Yes	0	0	0	—	—
Hypertension					
No	341	10,573	32.3	1 (reference)	1 (reference)
Yes	610	8,938	68.2	2.15 (1.88–2.46)***	1.05 (0.90–1.24)
Hyperlipidemia					
No	736	14,999	49.1	1 (reference)	1 (reference)
Yes	215	4,512	47.7	0.98 (0.84–1.14)	0.75 (0.64–0.88)***
Pulmonary embolism					
No	951	19,511	48.7	1 (reference)	1 (reference)
Yes	0	0	0	—	—
Depression					
No	938	19,143	49	1 (reference)	1 (reference)
Yes	13	368	35.3	0.73 (0.42–1.26)	0.53 (0.30–0.92)*
Stroke					
No	803	17,989	44.6	1 (reference)	1 (reference)
Yes	148	1,522	97.2	2.21 (1.85–2.64)***	1.36 (1.13–1.64)**
Heart disease					
No	476	12,370	38.5	1 (reference)	1 (reference)
Yes	475	7,141	66.5	1.77 (1.55–2.01)***	1.19 (1.03–1.38)*
Anxiety					
No	652	13,810	47.2	1 (reference)	1 (reference)
Yes	299	5,701	52.4	1.12 (0.98–1.29)	1.04 (0.89–1.21)
Smoking					
Tobacco dependence					
No	949	19,430	48.8	1 (reference)	1 (reference)
Yes	2	81	24.7	0.52 (0.13–2.10)	0.66 (0.16–2.67)
Tobacco use disorder complicating pregnancy					
No	951	19,511	48.7	1 (reference)	1 (reference)
Yes	0	0	0	—	—
Medication					
LABA					
Non-use	845	16,762	50.4	1 (references)	1 (reference)
Use	106	2,749	38.6	0.76 (0.62–0.93)**	0.70 (0.52–0.94)*
LAMA					
Non-use	914	18,744	48.8	1 (reference)	1 (reference)
Use	37	767	48.2	0.99 (0.71–1.38)	0.85 (0.59–1.22)
SABA					
Non-use	569	13,103	43.4	1 (reference)	1 (reference)
Use	382	6,408	59.6	1.37 (1.20–1.56)***	0.98 (0.79–1.21)
SAMA					
Non-use	657	15,329	42.9	1 (reference)	1 (reference)
Use	294	4,182	70.3	1.64 (1.43–1.88)***	1.22 (0.98–1.53)
ICSs					
Non-use	775	15,117	51.3	1 (reference)	1 (reference)
Use	176	4,394	40.1	0.77 (0.66–0.91)**	0.88 (0.69–1.11)
OSs					
Non-use	120	747	160.6	1 (reference)	1 (reference)
Use	831	18,764	44.3	0.26 (0.22–0.32)***	0.35 (0.29–0.44)***
Leukotriene antagonist					
Non-use	920	18,440	49.9	1 (reference)	1 (reference)
Use	31	1,071	28.9	0.57 (0.40–0.82)**	0.79 (0.19–3.24)
Montelukast					
Non-use	922	18,466	49.9	1 (reference)	1 (reference)
Use	29	1,045	27.8	0.55 (0.38–0.80)**	1.09 (0.25–4.65)
Alprazolam					
Non-use	646	11,677	55.3	1 (reference)	1 (reference)
Use	305	7,834	38.9	0.70 (0.61–0.80)***	0.73 (0.63–0.85)***
Fluoxetine					
Non-use	951	19,511	48.7	1 (reference)	1 (reference)
Use	0	0	0	—	—
Fludiazepam					
Non-use	797	15,575	51.2	1 (reference)	1 (reference)
Use	154	3,936	39.1	0.75 (0.63–0.90)**	0.79 (0.66–0.95)*

BCAS cohort, bronchiectasis–asthma combination cohort; COPD, chronic obstructive pulmonary disease; LABAs/LAMAs, long-acting beta2 agonist/muscarinic antagonist; SABAs/SAMAs, short-acting beta2 agonist/muscarinic antagonist; ICSs, inhaled corticosteroids; OSs, oral steroids; PY, person-years; IR, incidence rate per 1,000 person-years; HR, hazard ratio; CI, confidence interval; HR, adjusted for BCAS cohort, gender, age, comorbidities, and medication use.

—Unable to calculate because there are few or no events in the with and without BCAS cohort.

*p < 0.05, **p < 0.01, ***p < 0.001.

### Comparison of Non-BCAS and BCAS Cohorts

**FIGURE 1:**
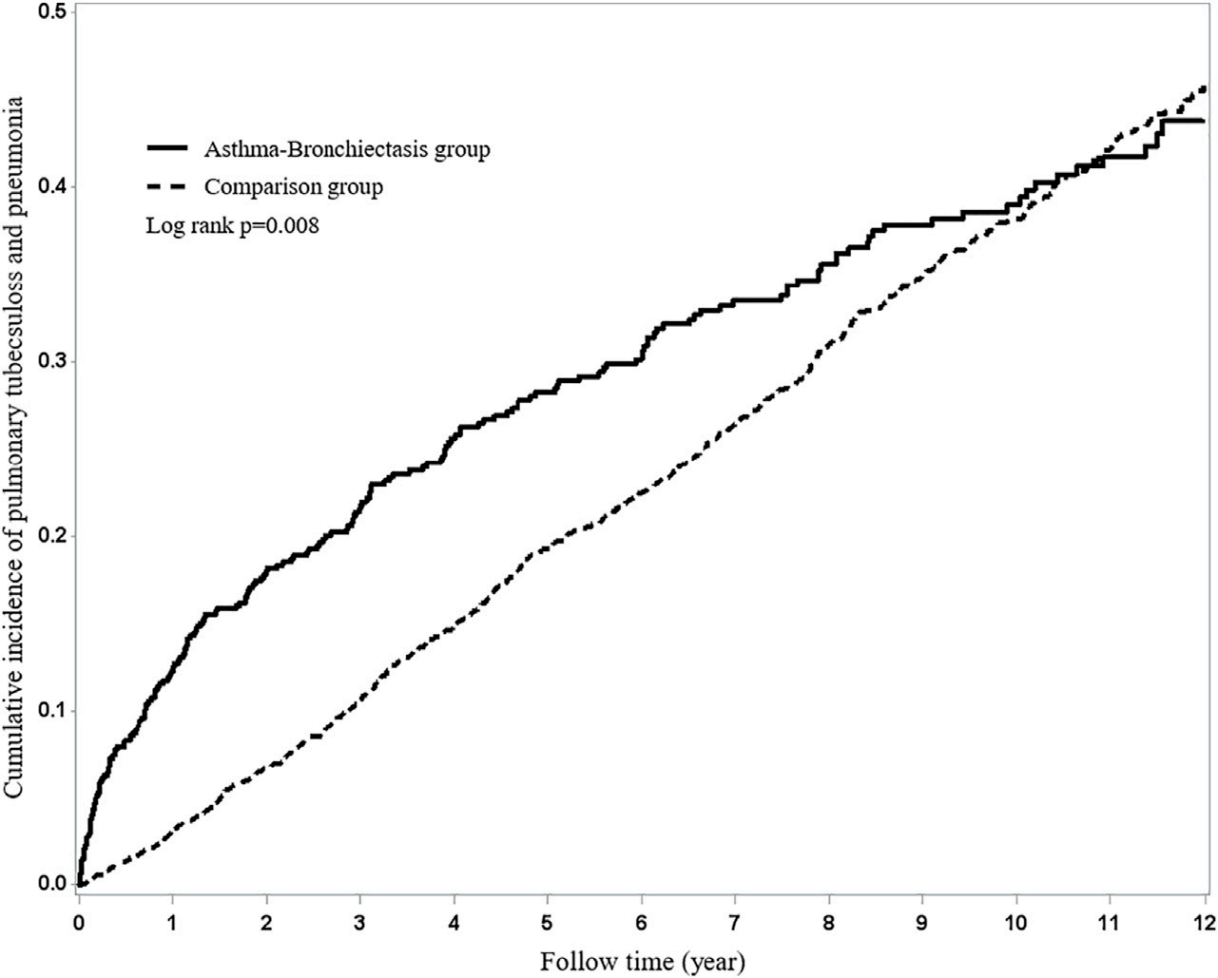
Using Kaplan–Meier survival statistics, the crude overall survival curves with and without bronchiectasis–asthma cohort are shown (log-rank *p* < 0.0001).

**FIGURE 2:**
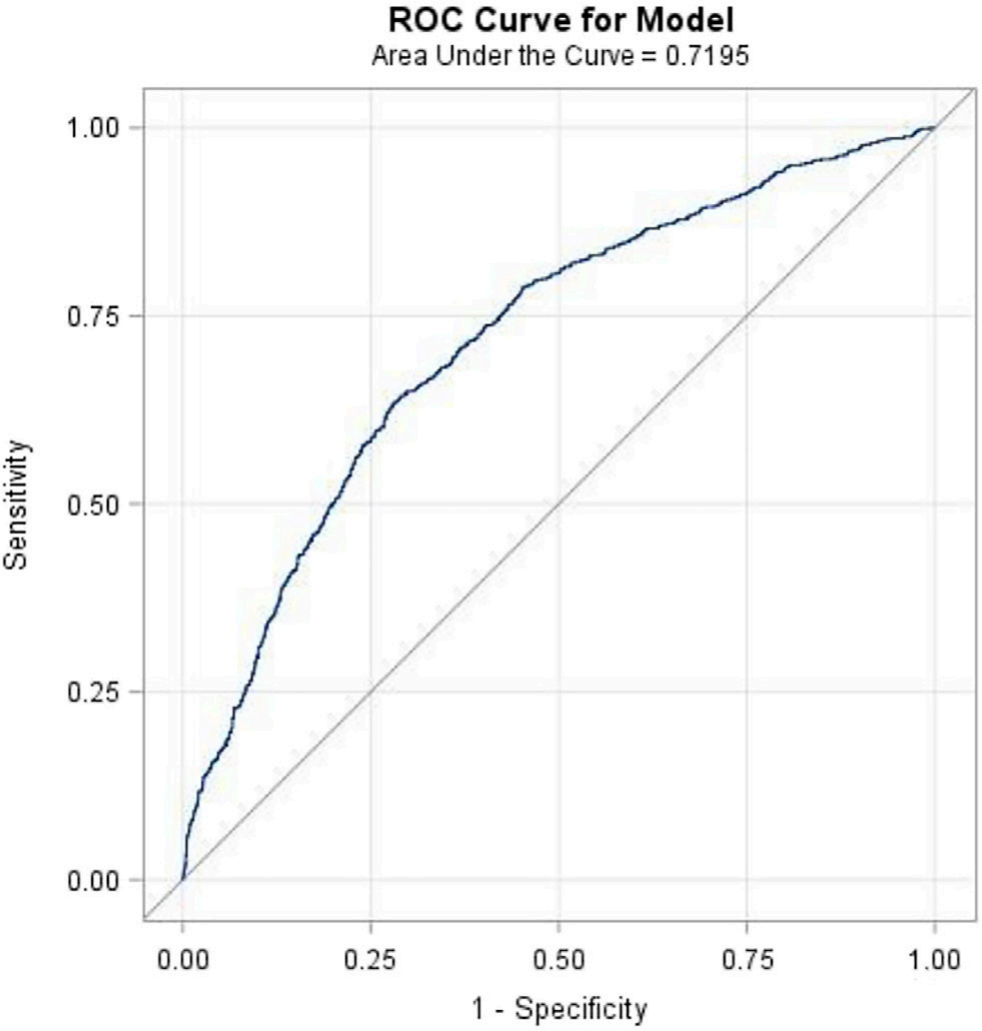
The area under the curve (AUC) for bronchiectasis-asthma cohort applied to predict pulmonary tuberculosis or pneumonia.

The results of the study revealed that 208 patients in the BCAS cohort and 743 patients in the non-BCAS cohort received a diagnosis of PTB or pneumonia (Table 3). After adjustment for age, comorbidities, and medications, women (aHR = 1.67, 95% CI = 1.33–2.09), those who were less than 35 years (aHR = 1.47, 95% CI = 1.25–1.72), those with COPD (aHR = 1.65, 95% CI = 1.35–2.02), those with diabetes (aHR = 1.64, 95% CI = 1.25–2.15), those with hypertension (aHR = 1.53, 95% CI = 1.25–1.87), those with heart disease (aHR = 1.64, 95% CI = 1.31–2.06), those with LABAs (aHR = 2.54, 95% CI = 1.63–3.69), those with ICSs (aHR = 2.00, 95% CI = 1.41–2.82), and those with OS (aHR = 1.38, 95% CI = 1.16–1.64) in the BCAS cohort were significantly more associated with risks of PTB or pneumonia than those in the non-BCAS cohort (Table 3). For current, recent, and past use, relative to the non-BCAS cohort, the risks of PTB or pneumonia were higher for those with SABAs (≤30 days), ICSs (≤30 days), and OSs (≤30 days), by 1.61 (95% CI = 1.31–1.96), 3.23 (95% CI = 1.96–5.29), and 1.76 (95% CI = 1.45–2.14), respectively. The risks of PTB or pneumonia were not associated with LAMAs (≤30 days) and SAMAs ≤30 days); the aHRs were 0.52 (0.14–1.84) and 1.45 (0.99–2.11), respectively (Table 4). A Kaplan–Meier analysis revealed statistically significant differences in the cumulative incidence of PTB or pneumonia between the BCAS and non-BCAS cohorts (log rank test: *p* = 0.008) (Figure 1). The area under curve for BCAS applied to predict PTB or pneumonia occurrence was 0.7195 (95% CI = 0.70–0.74) (Figure 2).

**TABLE 3:** Incidence rate and hazard ratio of pulmonary tuberculosis or pneumonia between the two cohorts stratified by gender, age, comorbidities, and medications after propensity matching.

	BCAS cohort						Crude HR (95% CI)	Adjusted HR (95% CI)
	No			Yes				
	Event	PY	IR	Event	PY	IR		
Gender								
Female	363	8,528	42.6	107	1987	53.9	1.26 (1.01–1.56)*	1.67 (1.33–2.09)***
Male	380	7,381	51.5	101	1,615	62.5	1.21 (0.97–1.50)	1.44 (1.15–1.80)**
Age								
<35	31	2,014	15.4	10	544	18.4	1.19 (0.58–2.43)	1.45 (0.65–3.26)
≥35	712	13,895	51.2	198	3,058	64.7	1.26 (1.07–1.47)**	1.47 (1.25–1.72)***
Comorbidity								
Non-tuberculous mycobacterium								
No	742	15,851	46.8	208	3,599	57.8	1.23 (1.05–1.43)**	1.54 (1.31–1.80)***
Yes	1	58	17.2	0	3	0	—	—
Rheumatoid arthritis								
No	715	15,363	46.5	200	3,489	57.3	1.22 (1.05–1.43)*	1.53 (1.30–1.79)***
Yes	28	546	51.3	8	133	60.2	1.34 (0.61–2.96)	2.37 (0.88–6.35)
Diffuse connective disease and Sjogren’s syndrome								
No	716	15,469	46.3	203	3,535	57.4	1.23 (1.05–1.44)**	1.56 (1.33–1.83)***
Yes	27	440	61.4	5	67	74.6	1.21 (0.46–3.19)	0.70 (0.22–2.21)
COPD								
No	291	8,098	35.9	81	1893	42.8	1.18 (0.92–1.51)	1.54 (1.19–1.98)***
Yes	452	7,811	57.9	127	1709	74.3	1.28 (1.05–1.56)*	1.65 (1.35–2.02)***
Diabetes								
No	478	11,706	40.8	135	2,733	49.4	1.20 (0.99–1.46)	1.50 (1.23–1.83)***
Yes	265	4,203	63.1	73	869	84	1.32 (1.02–1.72)*	1.64 (1.25–2.15)***
Aspergillosis								
No	742	15,890	46.7	208	3,596	57.8	1.23 (1.05–1.44)**	1.54 (1.31–1.80)***
Yes	1	19	52.6	0	6	0	—	—
Candidiasis								
No	743	15,909	46.7	208	3,602	57.7	1.23 (1.05–1.43)**	1.54 (1.31–1.80)***
Yes	0	0	0	0	0	0	—	—
Endemic mycoses								
No	743	15,909	46.7	208	3,602	57.7	1.23 (1.05–1.43)**	1.54 (1.31–1.80)***
Yes	0	0	0	0	0	0	—	—
Mounier-Kuhn								
No	743	15,909	46.7	208	3,602	57.7	1.23 (1.05–1.43)**	1.54 (1.31–1.80)***
Yes	0	0	0	0	0	0	—	—
Cystic fibrosis								
No	743	15,909	46.7	208	3,602	57.7	1.23 (1.05–1.43)**	1.54 (1.31–1.80)***
Yes	0	0	0	0	0	0	—	—
Hypertension								
No	258	8,484	30.4	83	2089	39.7	1.31 (1.02–1.67)*	1.58 (1.22–2.04)***
Yes	485	7,425	65.3	125	1,513	82.6	1.25 (1.03–1.52)*	1.53 (1.25–1.87)***
Hyperlipidemia								
No	566	12,143	46.6	170	2,857	59.5	1.27 (1.07–1.50)**	1.53 (1.29–1.83)***
Yes	177	3,766	47	38	745	51	1.09 (0.77–1.55)	1.56 (1.08–2.25)*
Pulmonary embolism								
No	743	15,909	46.7	208	3,602	57.7	1.23 (1.05–1.43)**	1.54 (1.31–1.80)***
Yes	0	0	0	0	0	0	—	—
Depression								
No	734	15,603	47	204	3,540	57.6	1.22 (1.04–1.42)*	1.52 (1.30–1.78)***
Yes	9	306	29.4	4	62	64.5	1.98 (0.61–6.44)	16.1 (0.95–27.5)
Stroke								
No	618	14,627	42.3	185	3,362	55	1.30 (1.10–1.53)**	1.55 (1.31–1.84)***
Yes	125	1,282	97.5	23	240	95.8	0.96 (0.62–1.51)	1.55 (0.95–2.52)
Heart disease								
No	364	9,913	36.7	112	2,457	45.6	1.24 (1.00–1.53)*	1.48 (1.19–1.84)***
Yes	379	5,996	63.2	96	1,145	83.8	1.33 (1.06–1.67)*	1.64 (1.31–2.06)***
Anxiety								
No	507	11,197	45.3	145	2,613	55.5	1.22 (1.01–1.47)*	1.55 (1.28–1.87)***
Yes	236	4,712	50.1	63	989	63.7	1.27 (0.96–1.68)	1.61 (1.21–2.14)**
Smoking								
Tobacco dependence								
No	741	15,849	46.8	208	3,581	58.1	1.23 (1.06–1.44)**	1.54 (1.31–1.80)***
Yes	2	60	33.3	0	21	0	—	—
Tobacco use disorder complicating pregnancy								
No	743	15,909	46.7	208	3,602	57.7	1.23 (1.05–1.43)**	1.54 (1.31–1.80)***
Yes	0	0	0	0	0	0	—	—
Medication								
LABA								
Non-use	673	13,817	48.7	172	2,945	58.4	1.19 (1.01–1.41)*	1.45 (1.22–1.72)***
Use	70	2,092	33.5	36	657	54.8	1.68 (1.12–2.52)*	2.54 (1.63–3.96)***
LAMA								
Non-use	716	15,351	46.6	198	3,392	58.4	1.24 (1.06–1.46)**	1.57 (1.34–1.84)***
Use	27	558	48.4	10	210	47.6	0.92 (0.44–1.92)	0.78 (0.33–1.82)
SABA								
Non-use	441	10,799	40.8	128	2,304	55.6	1.35 (1.11–1.65)**	1.61 (1.31–1.96)***
Use	302	5,110	59.1	80	1,298	61.6	1.03 (0.80–1.32)	1.39 (1.07–1.80)*
SAMA								
Non-use	506	12,567	40.3	151	2,762	54.7	1.35 (1.12–1.62)**	1.65 (1.37–1.98)***
Use	237	3,342	70.9	57	840	67.9	0.94 (0.70–1.26)	1.30 (0.96–1.77)
ICSs								
Non-use	621	12,584	49.3	154	2,534	60.8	1.22 (1.02–1.46)*	1.47 (1.22–1.75)***
Use	122	3,325	36.7	54	1,068	50.6	1.41 (1.02–1.94)*	2.00 (1.41–2.82)***
OSs								
Non-use	85	672	126.5	35	75	466.7	2.81 (1.88–4.21)***	3.99 (2.54–6.27)***
Use	658	15,237	43.2	173	3,527	49.1	1.14 (0.96–1.34)	1.38 (1.16–1.64)***
Leukotriene antagonist								
Non-use	724	15,167	47.7	196	3,273	59.9	1.25 (1.06–1.46)**	1.55 (1.32–1.81)***
Use	19	742	25.6	12	329	36.5	1.37 (0.66–2.84)	2.79 (1.07–7.27)*
Montelukast								
Non-use	725	15,170	47.8	197	3,296	59.8	1.24 (1.06–1.46)**	1.54 (1.31–1.80)***
Use	18	739	24.4	11	306	35.9	1.41 (0.66–3.00)	2.92 (1.11–7.64)*
Alprazolam								
Non-use	481	9,566	50.3	165	2,111	78.2	1.53 (1.28–1.83)***	1.86 (1.56–2.23)***
Use	262	6,343	41.3	43	1,491	28.8	0.68 (0.49–0.94)*	0.89 (0.64–1.24)
Fluoxetine								
Non-use	743	15,909	46.7	208	3,602	57.7	1.23 (1.05–1.43)**	1.54 (1.31–1.80)***
Use	0	0	0	0	0	0	—	—
Fludiazepam								
Non-use	619	12,723	48.7	178	2,852	62.4	1.27 (1.07–1.50)**	1.59 (1.34–1.89)***
Use	124	3,186	38.9	30	750	40	1.06 (0.71–1.58)	1.27 (0.84–1.91)

BCAS cohort, bronchiectasis–asthma combination cohort; COPD, chronic obstructive pulmonary disease; LABAs/LAMAs, long-acting beta2 agonist/muscarinic antagonist; SABAs/SAMAs, short-acting beta2 agonist/muscarinic antagonist; ICSs, inhaled corticosteroids; OSs, oral steroids; PY, person-years; IR, incidence rate per 1,000 person-years; HR, hazard ratio; CI, confidence interval; HR, adjusted for BCAS cohort, gender, age, comorbidities, and medication use.

—Unable to calculate because there are few or no events in the with and without BCAS cohort.

*p < 0.05, **p < 0.01, ***p < 0.001.

**TABLE 4:** Incidence rate and hazard ratio of pulmonary tuberculosis or pneumonia between the two cohorts stratified by the current, recent, and past use days.

	BCAS cohort						Crude HR (95% CI)	Adjusted HR (95% CI)
	No			Yes				
	Event	PY	IR	Event	PY	IR		
Drug-use days								
LABA								
Non-use	673	13,817	4.87	172	2,945	5.84	1.19 (1.01–1.41)*	1.45 (1.22–1.72)***
Current use (≤30 days)	38	615	6.18	25	180	13.89	2.22 (1.33–3.70)**	2.39 (1.31–4.34)**
Recent use (30–90 days)	6	113	5.31	2	22	9.09	2.82 (0.45–17.4)	--
Past use (>90 days)	26	1,364	1.91	9	455	1.98	1.12 (0.52–2.40)	3.72 (1.48–9.31)**
LAMA								
Non-use	716	15,351	4.66	198	3,392	5.84	1.24 (1.06–1.46)**	1.57 (1.34–1.84)***
Current use (≤30 days)	15	219	6.85	6	87	6.9	0.98 (0.38–2.54)	0.52 (0.14–1.84)
Recent use (30–90 days)	1	23	4.35	1	12	8.33	2.44 (0.15–39.7)	—
Past use (>90 days)	11	316	3.48	3	111	2.7	0.73 (0.20–2.68)	—
SABA								
Non-use	79	3,281	2.41	20	880	2.27	0.96 (0.59–1.58)	1.51 (0.90–2.55)
Current use (≤30 days)	441	10,799	4.08	128	2,304	5.56	1.35 (1.11–1.65)**	1.61 1.31–1.96)***
Recent use (30–90 days)	208	1,632	12.75	58	374	15.51	1.12 (0.84–1.51)	1.31 (0.94–1.82)
Past use (>90 days)	15	197	7.61	2	44	4.55	0.60 (0.13–2.64)	11.1 (0.56–21.8)
SAMA								
Non-use	506	12,567	4.03	151	2,762	5.47	1.35 (1.12–1.62)**	1.65 (1.37–1.98)***
Current use (≤30 days)	170	1,289	13.19	42	257	16.34	1.20 (0.86–1.69)	1.45 (0.99–2.11)
Recent use (30–90 days)	9	105	8.57	1	26	3.85	0.44 (0.05–3.54)	—
Past use (>90 days)	58	1,948	2.98	14	557	2.51	0.86 (0.48–1.55)	1.42 (0.76–2.65)
ICSs								
Non-use	621	12,584	4.93	154	2,534	6.08	1.22 (1.02–1.46)*	1.47 (1.22–1.75)***
Current use (≤30 days)	63	740	8.51	35	198	17.68	2.04 (1.34–3.10)***	3.23 (1.96–5.29)***
Recent use (30–90 days)	5	127	3.94	1	30	3.33	0.89 (0.10–8.04)	—
Past use (>90 days)	54	2,458	2.2	18	840	2.14	1.03 (0.60–1.77)	1.77 (0.98–3.20)
OSs								
Non-use	85	672	12.65	35	75	46.67	2.81 (1.88–4.21)***	3.99 (2.54–6.27)***
Current use (≤30 days)	441	4,529	9.74	152	1,039	14.63	1.52 (1.27–1.83)***	1.76 (1.45–2.14)***
Recent use (30–90 days)	45	1,307	3.44	4	381	1.05	0.30 (0.10–0.83)*	0.66 (0.23–1.93)
Past use (>90 days)	172	9,401	1.83	17	2,107	0.81	0.44 (0.26–0.72)**	0.63 (0.38–1.04)
Leukotriene antagonist								
Non-use	724	15,167	4.77	196	3,273	5.99	1.25 (1.06–1.46)**	1.55 (1.32–1.81)***
Current use (≤30 days)	6	56	10.71	7	38	18.42	1.53 (0.51–4.58)	—
Recent use (30–90 days)	2	35	5.71	1	6	16.67	1.81 (0.16–20.5)	—
Past use (>90 days)	11	651	1.69	4	285	1.4	0.80 (0.25–2.52)	1.56 (0.21–11.1)
Montelukast								
Non-use	725	15,170	4.78	197	3,296	5.98	1.24 (1.06–1.46)**	1.54 (1.31–1.80)***
Current use (≤30 days)	5	53	9.43	7	38	18.42	1.66 (0.52–5.27)	—
Recent use (30–90 days)	2	35	5.71	1	6	16.67	1.81 (0.16–20.5)	—
Past use (>90 days)	11	651	1.69	3	262	1.15	0.66 (0.18–2.39)	
Alprazolam								
Non-use	481	9,566	5.03	165	2,111	7.82	1.53 (1.28–1.83)***	1.86 (1.56–2.23)***
Current use (≤30 days)	102	1,253	8.14	27	228	11.84	1.51 (0.98–2.31)	1.73 (1.08–2.75)*
Recent use (30–90 days)	16	371	4.31	0	100	0	—	—
Past use (>90 days)	144	4,719	3.05	16	1,163	1.38	0.44 (0.26–0.74)**	0.59 (0.35–1.01)
Fluoxetine								
Non-use	743	15,909	4.67	208	3,602	5.77	1.23 (1.05–1.43)**	1.54 (1.31–1.80)***
Current use (≤30 days)	0	0	0	0	0	0	—	—
Recent use (30–90 days)	0	0	0	0	0	0	—	—
Past use (>90 days)	0	0	0	0	0	0	—	—
Fludiazepam								
Non-use	619	12,722	4.87	178	2,852	6.24	1.27 (1.07–1.50)**	1.59 (1.34–1.89)***
Current use (≤30 days)	20	268	7.46	10	79	12.66	1.69 (0.79–3.64)	7.48 (1.93–28.9)**
Recent use (30–90 days)	5	67	7.46	2	48	4.17	0.56 (0.10–2.93)	—
Past use (>90 days)	99	2,852	3.47	18	623	2.89	0.86 (0.52–1.42)	0.97 (0.58–1.63)

BCAS cohort: bronchiectasis–asthma combination cohort; COPD, chronic obstructive pulmonary disease; LABAs/LAMAs, long-acting beta2 agonist/muscarinic antagonist; SABAs/SAMAs: short-acting beta2 agonist/muscarinic antagonist; ICSs, inhaled corticosteroids; OSs, oral steroids; PY, person-years; IR, incidence rate per 100 person-years; HR, hazard ratio; CI, confidence interval; HR, adjusted for BCAS cohort, gender, age, comorbidities, and medication use.

—Unable to calculate because of there are few or no events in the with and without BCAS cohort.

a *p* < 0.05, **p < 0.01, ***p < 0.001.

### Propensity Matching

Before the experiment of propensity score matching, we performed a logistic regression to calculate the propensity score. Then, we estimated the probability of the BCAS cohort on the basis of the baseline variables, namely, index year, sex, age, history of comorbidities, and medicine. Finally, we performed multivariable Cox proportional hazards model stratification on the non-BCAS cohort to estimate the risks of PTB or pneumonia in the two cohorts.

### Immortal Time Bias

Users of drugs were defined as those who received at least one prescription for drugs (including bronchodilators, steroids, leukotriene receptor antagonist, montelukast, anti-arrhythmic, antidepressants, and antianxiety drugs) between the BCAS cohort diagnosis date and index date. The date of prescription and number of days supplied were identified. The use of drugs was approved in Taiwan in January 2000 and was placed on the listing of NHI drugs for reimbursement in February 2001. The drug users were defined as patients who received prescription for >28 days and after index date, and those who did not receive drug prescription were classified as non-users of drugs. The immortal time bias is eliminated. Any drugs prescribed after index date and before the endpoints for the subjects were considered as exposure.

The Taiwan government launched the pay-for-performance of asthma (asthma P4P program) in 2001 to encourage clinics and hospitals to provide patient-centered care and focus on disease management. This program includes initial visit for new patients, outpatient care, hospitalization, first prescription, emergency visits, and drug refill prescription (codes for disease management and care fees: P1612C, P1613C, P1614B, and P1615C, https://www.nhi.gov.tw/Content-). The initial visit for new patients and first prescription could help us to confirm if the patients are alive or not. These strict policies help us to eliminate the immortal time bias in this study (Suissa and Ernst, 2020).

### Validation of BCAS

One NHIRD study achieved high-sensitivity diagnosis of asthma with up to 92.0% accuracy on the basis of the clinical manifestation of asthma, family history of asthma, chest-X ray (CXR), and biochemistry and pulmonary function tests (Su et al., 2018). In Taiwan, the presence of bronchiectasis was confirmed based on the following high-resolution computed tomography (HRCT) criteria: 1) lack of tapering in the bronchi; 2) dilation of the bronchi where the internal diameter was larger than that of the adjacent pulmonary artery (e.g., broncho–arterial ratios >1); or 3) visualization of the peripheral bronchi within 1 cm of the costal pleural surface or the adjacent mediastinal pleural surface (Hill et al., 2019; Huang et al., 2020). Huang et al. (2020) used to diagnose bronchiectasis HRCT in 10,724 of the 15,729 patients (68.1%) and CXR in 5,005 of the 15,729 (31.9%) patients in a bronchiectasis cohort from the hospital database, and the average portion of patients having HRCT images increased annually (2002–2005: 43.1%, 2006–2010: 71.0%, and 2011–2016: 90.3%).

In this study, asthma was defined with ICD-9 CM code 493, combined with at least two prescriptions of anti-asthmatic drugs. Anti-asthmatic drugs include LABAs/LAMAs/SABAs/SAMAs/leukotriene receptor antagonist/montelukast/ICSs/OSs (Su et al., 2018; Yeh et al., 2019). Bronchiectasis was defined with ICD-9-CM code 494, combined with at least one CXR (including the posterior–anterior and lateral view) with computed tomography (CT) or two CXR with at least one inpatient visit or two outpatient visits. Moreover, the BCAS cohort receives procedures such as CXR (100%); CT-related test (83%); pulmonary function-related test (92.5%); asthma-related test and examination (96%); and medications such as the use of OSs (91.6%), SABA (34.8%), and ICSs (29.5%) (for code names, see Supplementary Table S1). These procedures, medications, and strategies help us to confirm the diagnosis of the BCAS cohort. Thus, subgroups of the BCAS cohort such as pure BCAS were derived from the bronchiectasis cohort presenting as patients having both components of the bronchiectasis and asthma in the general population (summary findings in Supplementary Table S2).

### After Matching Study Asthma Therapies on BCAS and Non-BCAS Cohorts

In the non-BCAS cohort (1 + 2 + 3 + 4 + 5 + 8), only 3 and 5 have the asthma component. The BCAS cohort (6 + 7), including 6 and 7, all have the asthma component; thus, the frequency of asthma therapies (LABAs, ICSs, leukotriene receptor antagonist, and montelukast use) in the BCAS cohort was higher than the non-BCAS cohort (Supplementary Figure S1).

### Validation of Tuberculosis and Use Interferon-Gamma for Early Detection of LTBI

During 1996–1999, tuberculosis and pneumonia were excluded before entry study, and only incident PTB or pneumonia were entered into the study (2000–2012, follow-up to 2013). The latency of the reactivation of the latent TB infection (LTBI) is about 2–5 years (Vynnycky and Fine, 1997). Thus, this wash out period (1996–1999) may be enough to help us to avoid these confounding factors.

Current methods for the diagnosis of LTBI are tuberculin skin test (TST) and interferon-gamma release assay (IGRA). The use of interferon for detecting LTBI was initiated from 2009 in Taiwan based on a published report (Chan et al., 2011; Lai et al., 2011). Also, like TST, IGRAs cannot distinguish between LTBI, active TB, or past infection. However, CXR, acid-fast stain, and culture for detecting active PTB were popular in Taiwan. Thus, the validation of PTB in NHIRD has high sensitivity (Su et al., 2016; Hsieh et al., 2019).

### Epidemiologic Studies Can Indicate Strengths of Associations Between PTB Pneumonia and the BCAS Cohort

This is fundamentally the same methodology as for a prospective cohort study, except that the retrospective study is performed *post-hoc*, looking back. Owing to the data of CXR, TST, and IGRA, smear or culture for bacteria and TB was unavailable in the NHIRD. As mentioned before, we excluded PTB or pneumonia before the entry study. However, post-PTB or post-pneumonia was a predisposing factor of airway disease (Bashir et al., 2016; Basham et al., 2021). Thus, we used the terms such as “BCAS cohort is associated with PTB or pneumonia” and “bronchodilators and steroids are associated with PTB or pneumonia in BCAS cohort” in this study. Therefore, it is important to recognize that causality cannot be established definitively through in our study; however, this study could provide important evidence to suggest information regarding the strength of an association between the BCAS cohort, bronchodilators, steroids, and infection such as PTB or pneumonia.

## Discussion

To the best of our knowledge, this study used the largest group to date to determine the effect of bronchodilators, steroids, antidepressant medicines, and BZDs on the risks of PTB or pneumonia among patients with the BCAS cohort. This study yielded three major findings as follows: 1) the risks of PTB or pneumonia were higher in the BCAS cohort than in the non-BCAS cohort; 2) compared to the non-BCAS cohort, in the BCAS cohort, bronchodilator (LABAs/SABAs) and steroid use was associated with risks of PTB or pneumonia, but BZDs and LAMAs/SAMAs were not; 3) current LABA/SABA and steroids use and BZDs use were associated with risks of PTB or pneumonia, but current LAMA/SAMA use was not associated with risks of PTB or pneumonia; and 4) the use of non-LABAs, non-OSs, and non-BZDs as reference 1 revealed that LABA, OS, and BZD use was associated with low risks of PTB or pneumonia.

The elevated neutrophil levels in sputum were associated with more exacerbations, low lung function, and greater duration and severity of bronchiectasis. Similarly, neutrophil elastase, derived from neutrophils, was found to be associated with an increased severity of bronchiectasis (Polverino et al., 2018; Mincham et al., 2021). The subgroups of the BCAS cohort such as BCAOS (7) include the asthma + bronchiectasis + COPD, and this triple component has complicated asthma, COPD, and bronchiectasis with high activity of the neutrophil (Polverino et al., 2018; Coman et al., 2018; Mihălț;an and Constantin, 2019; Mincham et al., 2021). The excessive activation of neutrophils results in the release of neutrophil elastase, leading to microenvironments that predispose to PTB or pneumonia among the BCAS cohort (Porto and Stein, 2016; Domon et al., 2018; Mihălț;an and Constantin, 2019). Thus, the BCAS cohort has a higher risk of PTB or pneumonia. Meanwhile, the subgroups of the BCAS cohort such as pure BCAS (6) with comorbidities such as diabetes, stroke, and heart disease also have higher risks (Porto and Stein, 2016; van der Meer et al., 2017; Domon et al., 2018).

Macrophage and monocytes expressed BZD-sensitive γ-aminobutyric acid type A (GABA_A_) receptors. The macrophage GABA_A_ receptor expression was regulated by bacterial Toll-like receptor agonists and cytokines, indicating an endogenous role in the immune response (Falcón et al., 2021). One previous study suggested that BZDs may negatively influence immune function *via* the activation of GABA_A_ receptors on immune cells such as activation or suppression of cytokine secretion and modification of cell proliferation, and GABA_A_ can even affect migration of the cells, thus interfering with macrophages/monocytes and impairing cytokine release, phagocytosis, and bacterial-killing capabilities (Sanders et al., 2013; Falcón et al., 2021). In addition, BZDs may increase the risk of aspiration by decreasing lower esophageal sphincter pressure (Obiora et al., 2013; Chang et al., 2016). Therefore, BZDs might have predicted the outcome in the analysis, and these drugs were entered into the analysis to start with (Huang et al., 2021).

Patients with non-cystic fibrosis bronchiectasis are at increased risk for depression and anxiety. Untreated and undetected depressive/anxiety symptoms may increase physical disability, morbidity, and health care utilization (Wynne et al., 2020). BZDs play an auxiliary role in the management of bronchiectasis especially in the late course of this disease (Kapnadak et al., 2020). In this study, the use of non-LABAs, non-OSs, and non-BZDSs as reference 1 revealed that LABA, OS, and BZD use was associated with low risks of PTB or pneumonia. Another study reported that anxiety was associated with the risks of PTB or pneumonia in BCAS in combination with low HRQoL (Duko et al., 2015; Gao et al., 2018). Thus, relieving anxiety may have a benefit for the prevention of the risk of PTB or pneumonia in patients within the BCAS cohort (Lehrer et al., 2008; Patel et al., 2019). However, patients with current BZD use (≤30 days, BZDs) but not past use (>90 days) had increased risks of PTB or pneumonia.

As with any propensity analysis, hidden, impactful confounders are always a possibility, and these BZD drugs may simply point to a hidden confounding variable such as the comorbidity diabetes. It is to be noted that comorbid-related inflammatory cytokines and stimuli reduce GABA_A_ receptor expression on alveolar macrophage (Antony et al., 2010; Obiora et al., 2013). A reduction in the expression of the receptor target, by comorbid inflammation, would be expected to reduce the immune effects of BZDs, leading to risk of PTB or pneumonia (Sanders et al., 2013).

These findings imply that 1) the primary effect of the BCAS cohort such as triplet (7, BCAOS) was a critical factor of the PTB or pneumonia; 2) the BCAS cohort such as pure BCAS (6) with comorbidities such as diabetes, stroke, and heart disease might be the relevant factor in subsequent PTB or pneumonia; and 3) perhaps steroids and BZDs have synergistic effects on these factors, leading to PTB or pneumonia.

As such, protopathic bias is a systematic error that occurs when measured exposure status medications may be affected by the latent onset of the target outcome—PTB or pneumonia. The lag-time approach consists in excluding from exposure assessment (PTB or pneumonia) the period immediately preceding the outcome detection date (≤30 days) (Arfè and Corrao, 2016). In this study, we excluded the patients with PTB or pneumonia before they enter into analysis. However, we found that the past use of medications (>90 days) is not associated with the risk of PTB or pneumonia except LABAs. These findings indicated that the protopathic bias may be an explanation of the association between the current use (≤30 days) of SABAs, steroids, and BZDs and PTB or pneumonia. If we take this possible protopathic bias into account, perhaps, the primary BCAS, especially its comorbidities, were key factors for the incident of PTB or pneumonia, and medications play a role on the synergistic effects.

In summary, because of the risks of PTB or pneumonia, LABAs/SABAs, steroids, and BZDs may be used in the BCAS cohort after benefit evaluation. However, LAMAs/SAMAs are safe options for the management of the BCAS cohort. Notably, we must take possible protopathic bias into account when we explain these findings.

### Strength

The accuracy of medical records in the NHIRD is high, making it a valid resource for population-based research on PTB or pneumonia. Bronchodilator and steroid use in Taiwan follows international guidelines (Yeh et al., 2018). Patients who use steroids were associated with PTB. Because bronchiectasis, asthma, and COPD were validated in the previous study on the basis of the NHIRD, the BCAS cohort data in the NHIRD is reasonable (Su et al., 2018; Huang et al., 2020). Therefore, our method prevented potential bias. In addition, we followed-up with the patients from 2000 to 2013 to monitor the incidence of PTB or pneumonia in the BCAS cohort (Huang et al., 2020).

### Limitations

Possible limitations of this study are bias and confounding variables, such as confounding by indication, protopathic bias, and surveillance bias. We used several strategies to control for bias and confounding variables, such as the new user design (entry after cohort) and the propensity matching method. With this framework, the results are not as accurate as those of randomized control trials. Data on lifestyle changes (exercise and diet) in low-adherence and high-adherence patients are unavailable in the NHIRD. Interferon use was associated with PTB infection. Owning to the higher frequency use of steroids than the interferon use in BCAS cohort and non-BCAS cohort, we replace the immunosuppressant (including the interferon) with steroids for analysis. However, this is another limitation. Finally, not all patients received HRCT in this study but at least two CXR for comparison of the irreversible change of bronchial tree (e.g., tram-track opacities) in outpatient visit or hospitalization. These confounding factors may have led to some bias in this study.

## Conclusion

The LAMAs/SAMAs are relatively safe with respect to PTB or pneumonia risks, but LABAs/SABAs, steroids, and BZDs could be used after evaluation of the benefit for BCAS cohort. However, we must take the possible protopathic bias into account.

## Summary

For pulmonary tuberculosis or pneumonia in predominant bronchiectasis–asthma, the long-acting/short-acting beta2 agonist, steroids, and benzodiazepines are to be used with caution; long-acting/short-acting muscarinic antagonists were relatively safe.

## Data Availability

The datasets presented in this article are not readily available because it is held by the Taiwan Ministry of Health and Welfare (MOHW). The Ministry of Health and Welfare must approve our application to access this data. Any researcher interested in accessing this dataset can submit an application form requesting access to the Ministry of Health and Welfare. Please contact the staff of MOHW (email: stcarolwu@mohw.gov.tw) for further assistance. The Taiwan Ministry of Health and Welfare is located in the following address: No.488, Sec. 6, Zhongxiao E. Rd., Nangang Dist., Taipei City 115, Taiwan (R.O.C.) (phone: +886-2-8590-6848). All relevant data are within the paper.

## Abbreviations

aHR, adjusted hazard ratio; PTB, pulmonary tuberculosis; BCAS, bronchiectasis–asthma combination; LABA, long-acting beta2 agonist; LAMA, long-acting muscarinic antagonist; SABA, short-acting beta2 agonist; SAMA, short-acting muscarinic antagonists; CI, confidence interval; ICSs, inhaled corticosteroids; OSs, oral steroids; BZDs, benzodiazepines; NHIRD, National Health Insurance Research Database; LHID, Longitudinal Health Insurance Database; ICD-9-CM, International Classification of Diseases, Ninth revision, Clinical Modification.
