# Supplementary material for: Asthma Therapies on Pulmonary Tuberculosis Pneumonia in Predominant Bronchiectasis–Asthma Combination
Source: Front Pharmacol. 2022 Mar 30;13:790031. doi: 10.3389/fphar.2022.790031 (PMC9006509; doi:10.3389/fphar.2022.790031)
Supplement: Supplementary file 1 [file Table1.DOCX]

| COMORBIDITIES | | |
| --- | --- | --- |
|  | **ICD-9CM** | **FULL NAMES** |
|  | 0.31 | NTM  Nontuberculous mycobacteria |
|  | 714.0 714.30 714.33 | rheumatoid arthritis |
|  | 710 | diffuse connective disease and Sjogren’s syndrome |
|  | 491, 492, and 496 | chronic obstruction pulmonary disease |
|  | 250 | diabetes |
|  | 117.3 | aspergillosis |
|  | 112.4 and 112.5 | candiasis |
|  | 114.0, 114.3, 114.4, and 114.5 | endemic mycoses |
|  | 748.3 | Mounier-Kuhn |
|  | 277.0 | cystic fibrosis |
|  | 401-405 | hypertension |
|  | 272.0-272.4 | hyperlipidemia |
|  | 415.1 | pulmonary embolism |
|  | 311 | depression |
|  | 433, 434, 435, and 436 | stroke |
|  | 410-414,425-429 | heart diseases |
|  | 300 | anxiety |
|  | 305.1, 305.11, 305.12, and 305.13 | tobacco dependence |
|  | 649.01 | tobacco use disorder complicating pregnancy |
| DRUGS (MEDICATIONS) | | |
|  | **ATC** | **FULL NAMES** |
| BRONCHODILAORS | R03AC12  R03AC13 | LABAs  long-acting beta2 agonists  R03AC12 ([Salmeterol](https://en.wikipedia.org/wiki/Salmeterol))  R03AC13 ([Formotero](https://en.wikipedia.org/wiki/Formoterol)l) |
|  | R03BB04 | LAMAs  long-acting muscarinic antagonists  R03BB04  [Tiotropium bromide](https://en.wikipedia.org/wiki/Tiotropium_bromide) |
|  | R03AC02,  R03AC03  R03AC04 | SABAs  short-acting beta2 agonists  R03AC02 [Salbutamol](https://en.wikipedia.org/wiki/Salbutamol)  R03AC03 [Terbutaline](https://en.wikipedia.org/wiki/Terbutaline)  R03AC04 [Fenoterol](https://en.wikipedia.org/wiki/Fenoterol) |
|  | R03BB01 | SAMAs  short-acting muscarinic antagonists  R03BB01  [Ipratropium bromide](https://en.wikipedia.org/wiki/Ipratropium_bromide) |
| STEROIDS | R03BA01, R03BA02,  R03BA05,  R03BA08 | ICSs  inhaled corticosteroids  R03BA01 [Beclometasone](https://en.wikipedia.org/wiki/Beclometasone)  R03BA02 [Budesonide](https://en.wikipedia.org/wiki/Budesonide)  R03BA05 Fluticasone  R03BA08 [Ciclesonide](https://en.wikipedia.org/wiki/Ciclesonide) |
|  | D07AC15, D07AC17, H02AB02, H02AB04, H02AB06,  H02AB08  R01AD05, S01BA02, | OSs  oral steroids  D07AC15 [Beclometasone](https://en.wikipedia.org/wiki/Beclometasone) D07AC17 [Fluticasone](https://en.wikipedia.org/wiki/Fluticasone_propionate)  H02AB02 [Dexamethasone](https://en.wikipedia.org/wiki/Dexamethasone)  H02AB04 [Methylprednisolone](https://en.wikipedia.org/wiki/Methylprednisolone)  H02AB06 [Prednisolone](https://en.wikipedia.org/wiki/Prednisolone)  H02AB08 [Triamcinolone](https://en.wikipedia.org/wiki/Triamcinolone)   \| R01AD05 Budesonide \|  \| \| --- \| --- \|   S01BA02Hydrocortison |
| LEUKOTRIENE RECPTORANTAGONIST | R03DC | Leukotriene receptor antagonist |
|  | R03DC03 | Montelukast |
| ANTI-ARRHYTHMIC | C01B | Anti-arrhythmic drugs |
| ANTIDEPRESSANTS | N06AB03 | Fluoxetine |
| BENZODIAZEPINES (BZDs) | N05BA12 | Alprazolam |
|  | N05BA17 | Fludiazepam |
| PROCEDURES | | |
| CEST-X-RAY | 32001C- 32002C |  |
| CT-RELATED | 33070B, 33071B, 33072B, 33103B |  |
| PULMONARY FUNCTION-RELATED TEST | 17001C, 17002B, 17003C, 17006C, 17004B-17018B, 17019C,  17020B - 17021B |  |
| ASTHMA-RELATED TEST and EXAMINATIONS | 12031C, 30021C-30022C, 30005B-30006B, 30009B-30010B, 30023B-30024B |  |
