# Supplementary figures and images for: Asthma Therapies on Pulmonary Tuberculosis Pneumonia in Predominant Bronchiectasis–Asthma Combination
Source: Front Pharmacol. 2022 Mar 30;13:790031. doi: 10.3389/fphar.2022.790031 (PMC9006509; doi:10.3389/fphar.2022.790031)

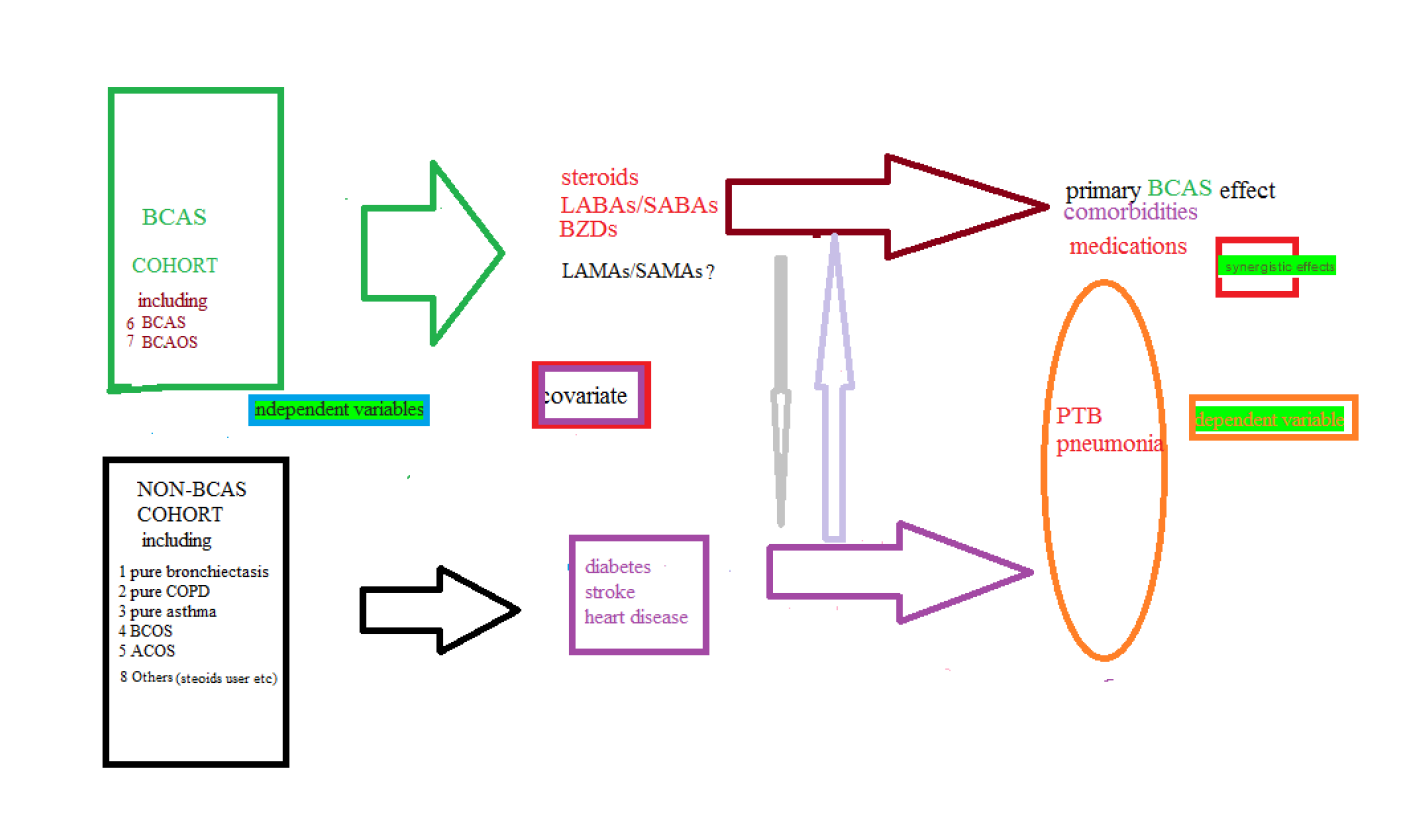

Supplement: Supplementary file 2 [file Image2.TIF]

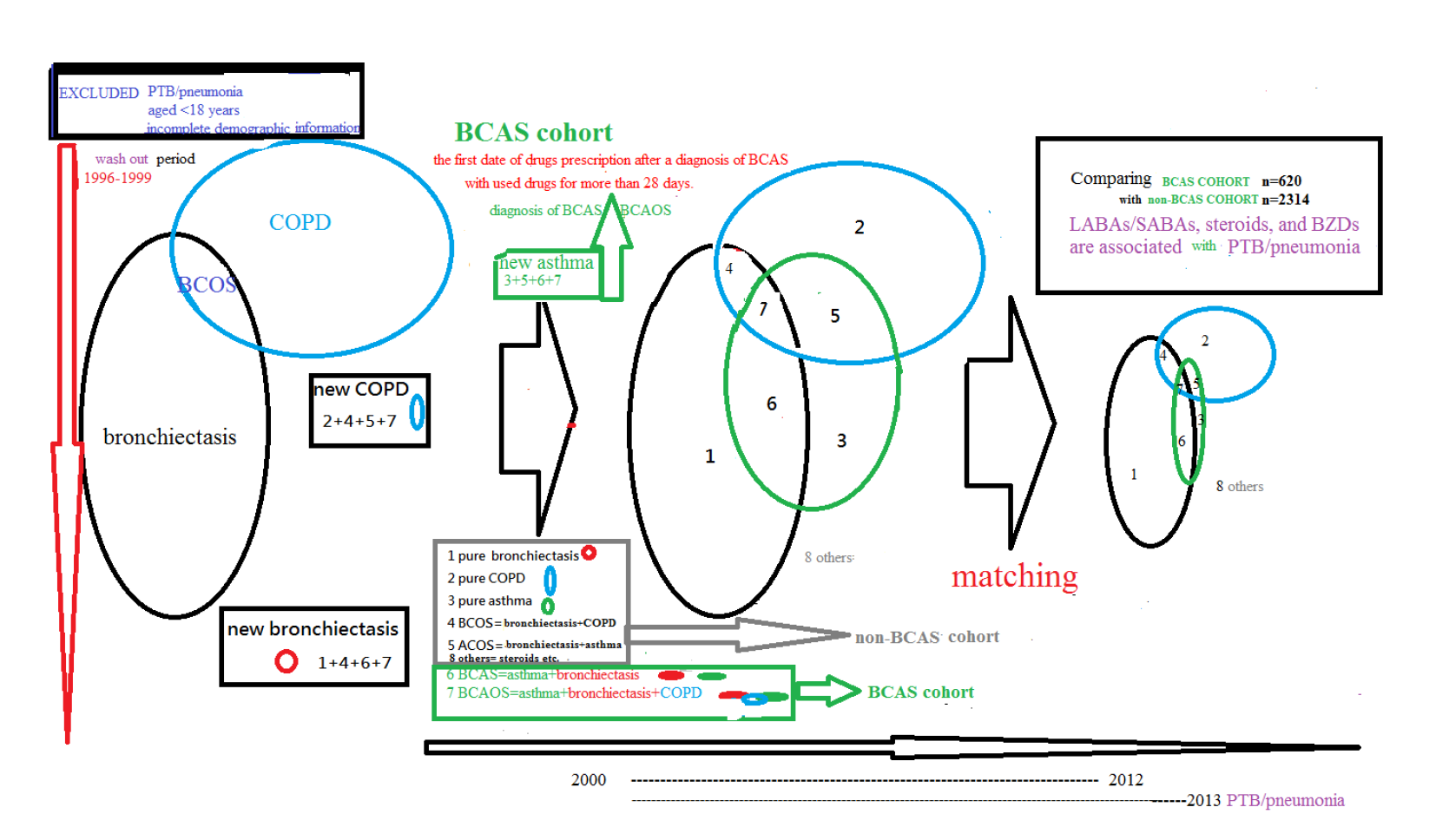

Supplement: Supplementary file 3 [file Image1.TIF]
