# Supplementary material for: Asthma Therapies on Pulmonary Tuberculosis Pneumonia in Predominant Bronchiectasis–Asthma Combination
Source: Front Pharmacol. 2022 Mar 30;13:790031. doi: 10.3389/fphar.2022.790031 (PMC9006509; doi:10.3389/fphar.2022.790031)
Supplement: Supplementary file 4 [file Table2.DOCX]

**Supplemental Table 2.**

|  | A | B | Past | Recent | Current |
| --- | --- | --- | --- | --- | --- |
| LABA | **-** | **+** | **+** |  | **+** |
| SABA | **0** | **+** | **0** | **0** | **+** |
| LAMA | **0** | **0** |  |  | **0** |
| SAMA | **0** | **0** | **0** |  | **0** |
| ICS | **0** | **+** | **0** |  | **+** |
| OS | **-** | **+** | **0** | **0** | **+** |
| Leukotriene antagonist | **0** | **+** | **0** |  |  |
| Montelukast | **0** | **+** |  |  |  |
| Alprazolam | **-** | **0** | **0** |  | **+** |
| Fludiazepam | **-** | **0** | **0** |  | **+** |

A: In general as no medicine as reference 1

B: The non-BCAS cohort as comparison cohort

-: Decreased risk; 0: Without effect; +: increased risk

LABAs/LAMAs: long-acting beta2 agonist/ muscarinic antagonist, SABAs/SAMAs: short-acting beta 2 agonist / muscarinic antagonist, inhaled corticosteroids: ICSs, and oral steroids; OSs
